# Co-Designing an Initiative to Increase Shared Access to Older Adults’ Patient Portals: Stakeholder Engagement

**DOI:** 10.2196/46146

**Published:** 2023-11-22

**Authors:** Vadim Dukhanin, Jennifer L Wolff, Liz Salmi, Kendall Harcourt, Deborah Wachenheim, Ira Byock, Matthew J Gonzales, Doug Niehus, Marianne Parshley, Caroline Reay, Sara Epstein, Supriya Mohile, Timothy W Farrell, Mark A Supiano, Anushka Jajodia, Catherine M DesRoches, The Shared Access Project Team

**Affiliations:** 1 Department of Health Policy and Management Johns Hopkins Bloomberg School of Public Health Baltimore, MD United States; 2 OpenNotes Beth Israel Deaconess Medical Center Boston, MA United States; 3 The Institute for Human Caring at Providence Gardena, CA United States; 4 Providence Medical Group Portland, OR United States; 5 Wilmot Cancer Institute University of Rochester Medical Center Rochester, NY United States; 6 Division of Geriatrics, Spencer Fox Eccles School of Medicine and the Center on Aging University of Utah Salt Lake City, UT United States; 7 Salt Lake City Geriatric Research, Education, and Clinical Center, Veterans Affairs Salt Lake City, UT United States; 8 School of Nursing Johns Hopkins University Baltimore, MD United States; 9 Department of Medicine Harvard Medical School Boston, MA United States; 10 See Authors’ Contributions

**Keywords:** patient portal, electronic health record, care partners, stakeholder engagement, patient engagement, human-centered design, mobile phone, design, older adults, digital platform, awareness, development, engagement, stakeholder, education

## Abstract

**Background:**

The patient portal is a widely available secure digital platform offered by care delivery organizations that enables patients to communicate electronically with clinicians and manage their care. Many organizations allow patients to authorize family members or friends—“care partners”—to share access to patient portal accounts, thus enabling care partners to receive their own identity credentials. Shared access facilitates trilateral information exchange among patients, clinicians, and care partners; however, uptake and awareness of this functionality are limited.

**Objective:**

We partnered with 3 health care organizations to co-design an initiative that aimed to increase shared access registration and use and that can be implemented using existing patient portals.

**Methods:**

In 2020, we undertook a rigorous selection process to identify 3 geographically diverse health care organizations that had engaged medical informatics teams and clinical champions within service delivery lines caring for older adults. We prioritized selecting organizations that serve racially and socioeconomically diverse populations and possess sophisticated reporting capabilities, a stable patient portal platform, a sufficient volume of older adult patients, and active patient and family advisory councils. Along with patients and care partners, clinicians, staff, and other stakeholders, the study team co-designed an initiative to increase the uptake of shared access guided by either an iterative, human-centered design process or rapid assessment procedures of stakeholders’ inputs.

**Results:**

Between February 2020 and April 2022, 73 stakeholder engagements were conducted with patients and care partners, clinicians and clinic staff, medical informatics teams, marketing and communications staff, and administrators, as well as with funders and thought leaders. We collected insights regarding (1) barriers to awareness, registration, and use of shared access; (2) features of consumer-facing educational materials to address identified barriers; (3) features of clinician- and staff-facing materials to address identified barriers; and (4) approaches to fit the initiative into current workflows. Using these inputs iteratively via a human-centered design process, we produced brochures and posters, co-designed organization-specific web pages detailing shared access registration processes, and developed clinician and staff talking points about shared access and staff tip sheets that outline shared access registration steps. Educational materials emphasized the slogan “People remember less than half of what their doctors say,” which was selected from 9 candidate alternatives as resonating best with the full range of the initiative’s stakeholders. The materials were accompanied by implementation toolkits specifying and reinforcing workflows involving both in-person and telehealth visits.

**Conclusions:**

Meaningful and authentic stakeholder engagement allowed our deliberate, iterative, and human-centered co-design aimed at increasing the use of shared access. Our initiative has been launched as a part of a 12-month demonstration that will include quantitative and qualitative analysis of registration and use of shared access. Educational materials are publicly available at Coalition for Care Partners.

## Introduction

The patient portal is a secure digital platform widely available throughout care delivery, through which individual patients can access their electronic medical records, schedule appointments, view test results and prescribed medications, and securely exchange messages with clinicians [1]. In the United States, many health care organizations allow patients to “share access” to their patient portal account (also referred to as “proxy access”) by authorizing a person to access the patient portal via a registration process. This process grants individual login information to the authorized care partner (ie, family, friend, patient advocate, or others who partner with patients to comanage their care) [2,3]. Shared access allows care partners to bidirectionally interact with clinicians on the patient’s behalf and access information necessary for supporting the patient’s care needs while using their own identity credentials. Available evidence finds shared access is desired by patients [4-6] and is consistent with the principles of patient-centered and family-centered care [7,8]. The presence of cognitive impairment and dementia poses a situation where both patients and care partners may benefit from information, communication, and support provided by shared access [9]. More explicit and purposeful engagement of care partners via the patient portal holds promise for advancing clinical quality and patient safety by increasing the transparency, accuracy, and comprehensiveness of patient health information across settings of care. Clarifying and executing patient desires to involve one or more care partners provide greater legitimacy and convenience to care partners in health care interactions and facilitate stronger trilateral partnerships between patients, care partners, and health care professionals [10,11]. Early evidence suggests that shared access increases patient and care partner satisfaction with health care services and care partner confidence in their ability to comanage care [12,13].

Currently, uptake and awareness of shared portal access functionality are low [14-16], and those who are aware of this functionality report that navigating the registration process is difficult and cumbersome to complete [6,17,18]. As a result, care partners commonly resort to accessing the portal using the patient’s identity credentials [15,16,18-21]. While using the patient’s credentials may appear convenient [18], there are significant drawbacks. The lack of differentiated portal identity credentials obscures clinicians’ understanding of whether and when care partners are involved in electronic interactions and leaves care delivery organizations without the ability to discern when a care partner is messaging clinicians, responding to patient assessments, or uploading legal documents, such as advance directives [22]. Care partners’ reliance on proper identity credentials affords patients greater autonomy and control of what is being shared, with whom, and for how long [22].

Co-design and human-centered design (HCD) are participatory design approaches that both honor the wisdom of the people most likely affected by an issue. Participatory design approaches mandate that researchers and organizations focus on understanding “end users” and thus engage end users in problem-solving and the development of solutions through, in case of HCD, participating in rapid prototyping and iterative refinement [23]. The HCD process is intentionally inclusive and collaborative, approaching community members as experts in their own lived experiences [24], making it especially well-positioned for the promotion of equity and the inclusion of voices of those who are marginalized [25]. The participatory design approaches also promote ownership of identified outcomes, thereby increasing the likelihood that interventions will be accepted, implemented, and sustained [26-28]. Co-designing and HCD are increasingly being used in health care improvement and have been used to develop clinical trials as well as health programs, products, diagnostic disparities solutions, websites, and technologies [29-32].

We describe an initiative, undertaken in partnership with 3 health care organizations, aimed at overcoming awareness and registration barriers that have inhibited uptake and use of shared access to the patient portal. First, we describe insights regarding barriers to use of shared access that were identified via stakeholder engagements at the 3 health care organizations and that can be implemented using existing patient portals. Second, we report on our use of co-design and HCD processes and present developed consumer-, staff-, and clinician-facing educational materials that would be poised for widespread scaling of the initiative. All materials are publicly available at Coalition for Care Partners [33].

## Methods

### Partner and Clinic Selection

A rigorous selection process was undertaken to identify 3 health care organizations to participate in our initiative to increase the uptake of shared access. This process initially drew upon a rich database, maintained by OpenNotes, the academic research initiative focused on open, transparent communication in health care [34]. OpenNotes’ database catalogs characteristics of more than 200 health care organizations that were among the early adopters of sharing medical visit notes with patients. We sought partners that varied by teaching status, electronic health record (EHR) vendor, rural or urban geography, and population diversity and considered 3 diverse organizations as a sufficient number for a scalable demonstration while accommodating available resources. The selection of candidate organizations was prioritized with engaged and innovative chief medical information officers, active Patient and Family Advisory Councils (PFACs), and stable patient portal and EHR platforms. Applying these criteria, we produced a short list of 13 organizations. Next, the study team gathered further information and assessed organizational interest in participating through informational interviews. We probed the suitability of candidate organizations by assessing these organizations’ patient portal registration rates, processes for granting shared access, capabilities for reporting their shared access use, the availability of a service delivery line in the organization that (1) cares for older adults, (2) employs a strong and enthusiastic clinical champion, (3) has sufficient volume of patients who are racially and socioeconomically diverse. While we aimed to partner with organizations that had implemented the patient portal from a variety of EHR vendors, the limited bandwidth on the part of the health care organizations due to the ongoing COVID-19 pandemic and the capacity to produce required portal use metrics prevented us from using this selection criterion. We ultimately identified 3 organizations that satisfied our inclusion criteria (Table 1).

**Table 1:** Overview of select health care organizations and their service delivery lines.

Organizational characteristics	Organization 1	Organization 2	Organization 3
Total number of physicians in the organization	1500+	10,000+	1200+
Total number of hospitals	6	50+	2
Profit status	Nonprofit	Nonprofit, church-operated	Nonprofit
Academic status	Major teaching	Minor teaching	Major teaching
Multistate organization	No	Yes, operates in 7 states	No
Number of clinics	1	3, in 1 city	2, in 1 location
Service delivery line, number of clinicians with established patient panel	Geriatric oncology, 8 clinicians	Primary care, 34 clinicians in total	Geriatrics and geriatric psychiatry, 19 clinicians in total
Service delivery line, number of staff	5	93	20
Service line, geographic location	Urban	Urban	Urban
Service line, patients, ages 65 years and older, total visits, annually	500+	30,000+	17,000+
Service line, patient and care partner engagement	PFAC^a^ of the clinic	PFAC at each clinic	Patient design studio, organization-wide
Organization electronic health record vendor type	Epic, since 2012	Epic, since 2011	Epic, since 2010
Health IT	MyChart, centralized health IT	MyChart, centralized health IT	MyChart, centralized health IT
Service line, patient portal registration	Approximately 85% of patients	Approximately 90% of patients	Approximately 95% of patients
Service line, shared access registration of care partners	Approximately 12% of patients have registered care partners	Approximately 3% of patients have registered care partners	Approximately 20% of patients have registered care partners

^a^PFAC: Patient and Family Advisory Council.

### Human-Centered Design

The HCD team was led by an HCD specialist (AJ) and a health care communications researcher who specializes in dissemination science (LS). We practiced the 5 stages of HCD: define, empathize, ideate, prototype, and test [35] (Table S1 in Multimedia Appendix 1). Below we illustrate the HCD activities that led to specifically the creation of our consumer-facing materials:

- Define (to describe the challenge based on what has been learned about stakeholders and context): literature review; discussions with our advisory panel (thought leaders), funders, and health care administrators; and inputs from patients and care partners.
- Empathize (to observe, engage with, and listen to stakeholders): interviews or focus groups with patients, care partners, clinicians, and staff to understand community culture and perspectives on factors driving low awareness and uptake of shared access.
- Ideate (to generate the broadest range of solution possibilities): our design team brainstormed on the story, messaging, and related visual elements of consumer-facing materials, and we solicited feedback from our stakeholders, including on the placement of materials and their compliance with organizational hygiene and marketing standards.
- Prototype (the process of producing an iterative generation of solutions for stakeholders to interact with or experience): we used a voting process during focus groups to elicit input from stakeholders on prototypes and slogans that will be used on both poster and brochure and convened training meetings and questions and answers sessions for staff and clinicians.
- Test (to solicit stakeholder feedback): we are now testing the educational materials in a 12-month demonstration that will include assessments and adjustments to address insights gained during interviews, focus groups, and open-ended surveys with stakeholders.

### Stakeholder Engagements

Between February 2020 and April 2022, a series of web-based focus groups and video calls were conducted with clinicians, clinic staff, patients and care partners, and organizational marketing and communications teams to understand the interests and expectations of each stakeholder group. We additionally administered a survey to a patient panel, solicited written responses from staff within front desk and medical informatics teams, and held regular meetings with clinic administrators. Engagements with thought leaders occurred quarterly at advisory panel meetings (listed in Multimedia Appendix 1) and at an annual site visit attended by representatives of private philanthropic foundations (funders).

Multiple stakeholder engagements were critical to developing an initiative that would ultimately resonate with each of our clinics while embracing universal elements that could be adapted by other organizations and for other populations. Table S1 in Multimedia Appendix 1 summarizes the distribution of 73 stakeholder engagements and illustrates how they correspond to the stages of HCD.

### Analysis of Inputs From Stakeholder Engagements

We used rapid assessment procedures to analyze and synthesize stakeholder input from different groups at our 3 health care organizations [36,37]. This flexible but rigorous approach to qualitative data analysis is appropriate for studies that are conducted over a relatively short time frame with a small number of specific research questions. The rapid assessment procedure, with its use of triangulation and iterative summaries, was selected as a complement to co-designing.

### Ethical Considerations

This study was reviewed and determined to be exempt by the Johns Hopkins Bloomberg School of Public Health Institutional Review Board (IRB number 19886).

## Results

### Overview

We organize the presentation of our results around four elements: (1) describing the service delivery lines that participate in the initiative; (2) providing insights on barriers to increased uptake and use of shared access by stakeholders that informed the initiative; (3) designing consumer-, staff-, and clinician-facing materials; and (4) aligning the initiative into existing clinic workflows. Stakeholder-identified barriers and features of the initiative are presented in Table 2.

**Table 2:** Stakeholder-identified barriers and features of the initiative informed by these barriers.

Insights on barriers to increased registration and use of shared access	Features of consumer-facing materials to address the barriers	Features of clinician- and staff-facing materials to address barriers	Enrolling the initiative within existing infrastructure
Cumbersome registration for both patients and staff Lack of awareness and knowledge Privacy concerns Worries about increased workload The digital divide: reaching patients with more limited health literacy or technological access and experience	Multiple modality (printed, QR code, telephone number, after-visit summary, and web page) Emphasizing the benefits of shared access Stressing that the patient is in control of granting shared access Including help desk contacts for support, if it exists Multilingual educational materials target consumers in general and not care partners specifically Materials use text but no human photographs	Talking points for clinicians and staff Tip sheets with resources and registration steps for staff Presentations to clinicians and staff on benefits Stressing clear lines of communication with patients and care partners as a benefit to clinicians and staff Smart phrase to generate text for the after-visit summary	“Light touch” to existing workflows of both in-person and telehealth visits Multiple touch points for receiving information Clinicians and staff have materials on hand to quickly answer patient questions

### Description of the Service Delivery Lines That Participate in the Initiative

Characteristics of the 3 organizations and service delivery lines are presented in Table 1. These organizations varied by size, operational features, and geography. Each organization had at least 10 years of experience with their patient portal and reported high portal registration rates (at least 85% of patients) and care partner shared access registration rates (2% to 21% of patients have 1 or more care partners with shared access) that were comparable to nationwide rates [21]. Participating service lines within each organization varied in size by number of clinicians (range 8 to 34), staff (range 5 to 93), patients served (range ~500+ to ~30,000+), clinical specialization (geriatric oncology, geriatrics, geriatric psychiatry, and primary care), and number of participating clinics (range 1 to 3).

### Insights on Barriers to Uptake and Use of Shared Access

Stakeholders provided insights on technology and workforce barriers that inhibited care partner registration for shared portal access at the 3 organizations (Table 2). Stakeholders were not asked to rank the barriers in terms of their importance. Cumbersome registration processes were named by patients and care partners, as well as by clinic staff and medical informatics teams. Where web-based registration is available, its awareness among patients and care partners was low. In-person registration processes commonly involve multiple steps. The requirement that both the care partner and patient be present in person to complete or need to fax privacy compliance forms posed challenges for staff, who are tasked with procuring and ensuring the form completion, as well as for patients and care partners. Stakeholders identified added difficulty when care partners were not established patients of the health care organization due to the necessity of setting up a new portal account, prior to being registered for shared access. Clinicians and staff reported that their lack of knowledge about the shared access registration process was a significant barrier. Because shared access registration is infrequent, clinic staff were described as having to “relearn” the process. Clinicians raised concerns about additional uncompensated work associated with increased messaging with care partners.

Other barriers noted by stakeholders originated from a lack of knowledge about what a person with shared access can and cannot do on the patient portal. Patients, clinicians, and staff raised patient privacy concerns and concerns for patient autonomy and noted the need for guidelines to clearly delineate best practices for workflows surrounding shared access registration and use. Finally, thought leaders, administrators, and patients raised concerns about diversity, equity, and inclusion regarding the potential disproportionate uptake of shared access among patients with more resources (digital divide) and a desire that shared access workflows be equitable, destigmatizing, and sensitive to the varied needs of all patients.

These barriers informed the co-designing of the initiative that relied on using existing patient portals. The co-design process also generated insights on barriers that our initiative was not able to address. We present the latter barriers in the discussion as limitations of the initiative.

### Designing Our Initiative

#### Overview

Stakeholder feedback guided all aspects of the initiative, which involved developing educational materials for each of our key groups to raise their awareness, communicate benefits, and explain and simplify shared access registration focusing on older adults. Here, we summarize how we approached (1) designing consumer-facing materials and (2) providing clinicians and staff with easily accessible information to overcome knowledge gaps. In addition, see Table 2 for the features of the initiative.

#### Designing Consumer-Facing Materials

Clinicians, patients, and thought leader stakeholders expressed the view that all materials should target consumers in general (as opposed to care partners specifically) to align with respect to patient privacy and autonomy, which was a central tenet of our design process. All educational materials were designed for customization to fit with each of our 3 partner organizations’ branding, color palettes, typefaces, and logos—to increase the likelihood, those materials would receive approval by site marketing and communications staff. Consumer-facing materials deliberately used text and did not include pictures of people. This was a design strategy, strongly endorsed by patients and care partners, to ensure the materials were broadly inclusive and did not inadvertently suggest that shared access was meant for patients of certain ages, genders, races, or ethnicities. For our texts, we used large types and accessible fonts.

The main slogan for our initiative, “What did the doctor say?” was selected, as it most resonated with patients and care partners in describing the benefits of using patient portals for remembering what happened during medical visits. The phrase “People remember less than half of what their doctors say” was based on the literature [38] and was endorsed by both patient and clinician audiences as a simple and relatable fact. Additionally, this slogan—initially applied to our initiative for older adults—was recognized as audience-neutral and thereby had the potential to be further expanded to additional audiences such as young adults. It was emphasized by one stakeholder from the marketing and communication group who reminded the study team that “the most successful messages are simple.”

**Figure 1:**
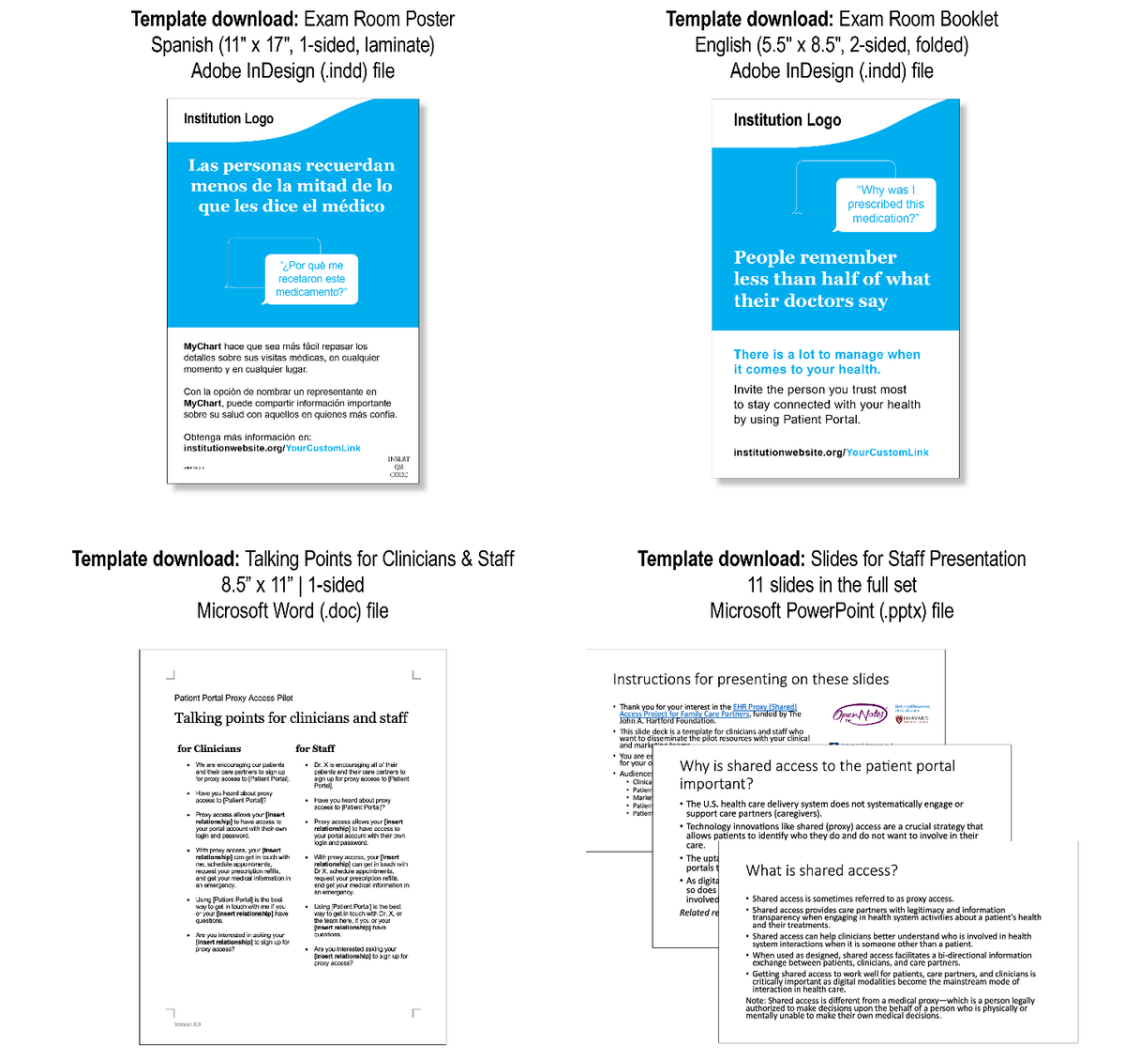
Educational materials and template downloads. Educational materials and templates are available for public use and download at Coalition for Care Partners.

Stakeholders endorsed the value of creating materials to serve multiple modalities. The printed materials—brochures and posters (see Figure 1 and Coalition for Care Partners [33] with publicly available unbranded materials)—included the organizational web page URL that provides additional information on the steps to assign shared access specific to each organization and a telephone number to get direct support from each organization’s patient portal helpline. Brochures included a QR code that can be scanned by a mobile phone and quickly bring visitors to the corresponding web page. The QR code feature was suggested by patients who said that they became more familiar with using QR codes after they had been popularized (and sometimes necessitated) throughout the COVID-19 pandemic at restaurants and other locations. The readability scores for the poster were Flesch reading ease of 50 and Flesch-Kincaid grade level of 9.9 and for the brochure were Flesch reading ease of 70 and Flesch-Kincaid grade level of 6.5.

#### Providing Clinicians and Staff With Easily Accessible Information

To address clinician and staff knowledge barriers, a tip sheet was developed to demystify registration processes, and talking points were created to use during conversations with patients and care partners and emphasize the convenience and benefits of shared access (Figure 1). To address clinician and staff concerns about increased workload, clinical champions and clinic leadership stressed the potential of shared access in streamlining communication and increasing efficiency, for example, by promoting electronic communications over telephone calls with care partners. The tip sheets, talking points, and in-service meetings emphasized the benefit of shared access in alleviating privacy concerns and promoting the use of proper identity credentials. It was stressed that shared access ensures that care partners are authorized by patients to electronically interact with clinicians. Embedded in the EHR, smart phrases help clinicians by automatically populating after-visit summaries with information about shared access registration and benefits.

### Aligning the Initiative Into Existing Clinic Workflows

**Figure 2:**
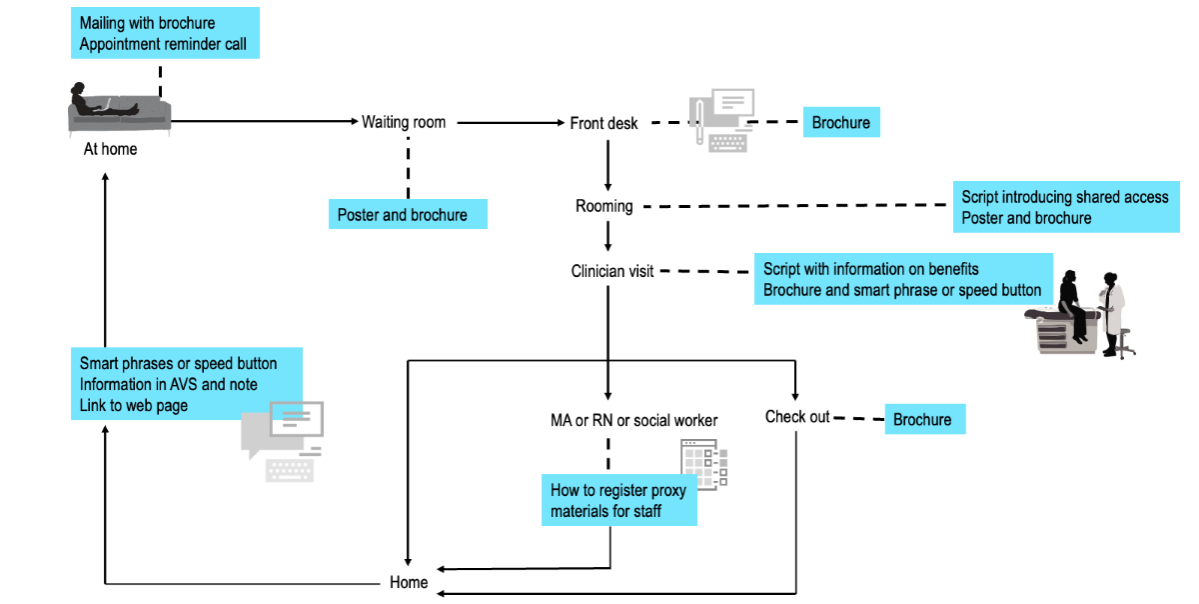
Multiple touch points and materials for introducing shared access. AVS: after-visit summary; MA: medical assistant, RN: registered nurse.

There was a strong consensus among stakeholders that the initiative should use multiple touch points (Figure 2 and Table 2) for broader reach and to reinforce awareness. For example, when the patient is at home, ahead of a visit, they may receive a brochure as a part of a new patient packet mailed to their home. The brochure explains shared access and its benefits and functionality, clarifies privacy, and connects to web-based instructions regarding registration processes with information about further registration support (eg, a helpline). Clinic staff may ask an older patient at appointment reminder calls if their care partner plans to join that appointment. At that time, if appropriate, additional information about shared access registration may be mentioned by the clinic staff. In clinic waiting rooms and exam rooms, posters reinforce the campaign message of “What did the doctor say?” Additionally, clinics are equipped with brochures (in English and Spanish) that are available for patients or care partners to take. These brochures can also be offered at the check-in desks by the staff during the check-in process.

As shown in Table 3, workflows were adapted to each clinic. At 3 of the 5 clinics, medical assistants raise the topic of shared access and offer brochures during rooming or in telehealth visit communication. At all clinics, clinicians are encouraged to raise and endorse the benefits of shared access and can offer patients a brochure or ask staff to provide the brochure to the patient or care partner before leaving the clinic. During in-person or telehealth visits, clinicians can insert a smart phrase in the patient’s medical record that prompts the inclusion of a 1-paragraph description about shared access in the patient’s after-visit summary and visit note. At telehealth visits, clinicians can share the electronic version of the brochure. Additionally, the smart phrase automatically includes a link to the organization’s web page with more details on shared access registration processes. At 2 of the 5 clinics, front-desk staff give patients and care partners the brochure at checkout. At 3 of the 5 clinics, patient service specialists are empowered to initiate conversations about shared access during telephone calls.

At a patient’s request, a medical assistant, registered nurse, or social worker can help the patient register their care partners for shared access while they are at the clinic after the visit. Alternatively, patients and care partners can register for shared access at home using the brochure, web page, and support telephone number named in the materials. Finally, after a visit, patients may receive an email notification about new documents on their portal that contains information about shared access with links to the organization’s web page and details about designating a care partner for shared access.

**Table 3:** Clinic staff and clinician roles in the initiative increasing the use of shared access and tailored to clinic workflows.

Staff	Clinic staff and clinician roles					
	Organization A			Organization B	Organization C	
	Clinic 1	Clinic 2	Clinic 3	Clinic 4	Clinic 5	
Front desk staff	No role	Offer brochure	Offer brochure	No role	No role	
Medical assistant	Initiate conversation while rooming and offer brochure	No role	No role	Initiate conversation while rooming and offer brochure	Initiate conversation while rooming and offer brochure	
Patient services specialist	Initiate conversation during telephone calls to the clinic	Initiate conversation during telephone calls to the clinic	Initiate conversation during telephone calls to the clinic	N/A^a^	N/A	
Clinician	Initiate or continue conversation with the patient, offer brochure, and include information in the after-visit summary	Initiate or continue conversation with the patient, offer brochure, and include information in the after-visit summary	Initiate or continue conversation with the patient, offer brochure, and include information in the after-visit summary	Initiate or continue conversation with the patient, offer brochure, and include information in the after-visit summary	Initiate or continue conversation with the patient, offer brochure, and include information in the after-visit summary	
Nurse navigator	N/A	N/A	N/A	Help patients and care partners with the sign-up	N/A	
Case manager or social worker	On referral, help patients and care partners proxies with the sign-up	No role	On referral, help patients and care partners proxies with the sign-up	N/A	Help patients and care partners with the sign-up	

^a^N/A: not applicable.

## Discussion

### Principal Findings

We present the main components of an initiative to increase awareness and use of shared access through the delivery of educational materials and implementation workflows that are poised for widespread scaling throughout mainstream care delivery. This initiative was informed by stakeholder engagements and co-design and HCD processes, during which we ideated and prototyped solutions to a series of practical questions: What are the barriers to increased use of shared access? How might these barriers be overcome? How might the initiative be implemented using the existing patient portal and current workflows? Our processes, iterations, and meaningful engagement of diverse stakeholder perspectives were essential to developing an initiative that would resonate with all stakeholders at each of our 3 health care organizations while also being broadly replicable and scalable. Our innovative and novel initiative addresses a crucial gap between the current low uptake of shared access and the potential for care partners of older adults to widely and beneficially use shared access [21].

### Future Directions

Our initiative has several immediate future directions. First, as educational materials were only offered in English and Spanish, we plan to offer materials in additional languages commonly spoken in the local communities. The possibility of new workflows for patients with limited English proficiency and their English-speaking care partners will be explored, including the use of interpreters for shared access registration. Second, the research team had hoped to implement the use of a video aid about shared access and registration instructions for clinic waiting rooms; however, this was not feasible for a variety of logistical reasons related to clinic workflow and EHR vendor policies—we are committed to investigating this further. Additional next steps involve conducting a 12-month evaluation with ongoing assessments and potential corresponding adjustments and dissemination activities aimed toward scaling and spreading the initiative.

The ongoing COVID-19 pandemic created a significant strain on health care organizations, individual clinicians, and their staff, resulting in high levels of burnout and staffing turnover, and the 3 participating organizations were not immune to these effects. For instance, one of our partner clinics, a geriatric psychiatry clinic, had to withdraw from the testing phase of the initiative because of turnover. It was critical that we worked closely with clinician and staff stakeholders from the beginning to understand the challenges they faced and to create an initiative that was perceived as being feasible and worthwhile to implement in individual clinics, as interventions are unlikely to be successful if perceived as burdensome. Organizational leaders are keenly aware of the difficult conditions faced by clinicians and staff and may be reluctant to undertake an initiative that involves additional effort, especially giving increasing volumes of electronic messaging [39], even if stakeholders believe it is the right thing to do. However, the pandemic amplified the practical significance of this work due to the shift toward remote modalities and electronic engagement and the need to identify strategies to outreach the most vulnerable and disadvantaged patients. The pandemic fundamentally changed patient workflows, leading to the development of educational materials and implementation toolkits that will operate in post–COVID-19 environments. Further research demonstrating the value of shared access to clinicians and the return on investment for organizations would help ensure that improving care partner registration becomes part of an organization’s strategic plan.

We recognize that the evolution of health information technology and public policy will affect shared access to the patient portal in future years. The development and spread of efforts to facilitate more direct, systematic, and purposeful engagement of care partners through shared access are especially timely in the context of the 21st Century Cures Act [40]. As of October 6, 2022, the Final Rule implementing the Cures Act mandated health care providers give patients electronic access without charge or delay to all the information in their electronic medical records through patient portals or third-party smartphone apps [41]. The same information is shared with all those who have access to the patient portal such as care partners with registered shared access. This new rule expands the types, comprehensiveness, and timeliness of information available to patients and care partners through the patient portal [41]. These technological connections make it possible for people to access, merge, and share their health data with others more easily. However, for those concerned about data privacy, this change in policy signals a need for increased patient education on how to use third-party applications safely and securely. Another emerging technology is chatbots embedded in the patient portal. Chatbots could be used to provide instructions and support for patients interested in granting shared access to a care partner. As our study team thinks about adapting to these changing digital times, HCD best practices might be deployed to address the iterative and emerging needs of end users before making assumptions and implementing solutions that affect all stakeholders involved. We will remain focused on eliciting feedback from all stakeholder partners—patients, care partners, clinicians, clinic staff, administrative and medical informatics, marketing, and communications staff—on how we might best communicate the nuances of data and privacy concerns to patients as well to the care partners hoping to help on their behalf.

### Comparison to Prior Work

Shared access to the patient portal has been successfully used in pediatric care [42,43]. Pediatric shared access, especially for adolescents, presents challenges that are similar to shared access for older adults, including the need to ensure that confidential information is shared with proper protections. Nonetheless, many health care organizations successfully share information with pediatric patients and their proxies, suggesting that achieving wide awareness and use of shared access among care partners of adults is likewise feasible. However, pediatric shared access has unique challenges due to state-by-state regulations around information confidentiality that are not formally applicable to shared access for adults. Additionally, as patient portal use becomes a mainstream modality in health care interactions, we should prioritize efforts to achieve digital health equity for older adults, many of whom are frequent health care users and least likely to be able to use complex patient portals [4,22,44,45].

It is worth noting that stakeholders discussed barriers to the optimal use of shared access that were beyond the scope of our intervention to address. Low staffing and staffing turnover were repeatedly voiced by our partner organizations as significant barriers. These also included the use of the term “proxy access” (used in EHRs instead of “shared access”)—many felt this was too easily confused with “health care proxy.” Clinicians also identified the need for paid caregivers (eg, staff in assisted living facilities) to have shared access to the patient portal. Stakeholders requested a set of norms of expectations, both under ethical and legal domains, for those with shared access. For example, clinicians reported being unsure of how to handle a situation in which multiple individuals, potentially with different perspectives on the patient’s care, might need access to the portal for the same patient. They further expressed concerns about sensitive information, such as test results, that might be seen by a care partner before the patient themselves or before the patient discusses information with their clinician. Finally, all stakeholders wanted more granular privacy controls on the portal to allow patients to select the information and functionalities that those whom they have authorized for shared access could view and use. For example, some older adults may prefer to allow an adult child to view information related to an orthopedic issue but to maintain the privacy of mental health visit notes. Addressing the broader functionality of shared access was not the focus of our initiative and would require larger organizational policy and EHR vendor changes.

### Strengths and Limitations

This work faces several limitations. We set a high bar for the selection of health care organizations; thus, our experiences will undoubtedly not represent the broader degree of readiness and enthusiasm for shared access. This was a deliberate choice to identify what it takes in a setting that has minimal resistance to innovation. However, the characteristics of the service delivery lines and the materials that have been developed create an opportunity to scale the initiative within and across a range of health care organizations. Our approach to analyzing stakeholder input was based on rapid assessment procedures and did not use formal qualitative methods as we had a limited timeline and have been focused on identifying the most salient and practical inputs on specific predefined questions. We collected insights on barriers to uptake and use of shared access specific to 3 health care organizations and co-designed the initiative that can be implemented using existing patient portals. With our use of the existing patient portals, we were not able to address all of the barriers raised. Thus, future research is needed to identify and solve the range of barriers faced by various other health care organizations. Likewise, developing solutions that modify how the patient portal or EHR systems function, for example, adding pop-ups or other alerts or implementing chatbots, was not within our scope and requires further exploration. Finally, the described initiative has not yet been fully tested and evaluated for its effectiveness and efficiency. However, we are sharing our processes, stakeholder inputs, and publicly available materials to expedite the spread and scaling of this initiative that our stakeholders consider as highly promising. In terms of our strengths, we had rigorously executed iterative co-design and HCD processes with a focus on implementation. We also consider that both focus on older adults, yet the versatility of consumer-facing materials allows for the initiative to be of interest of and be scaled among service delivery lines that do not provide care to older adults exclusively.

### Conclusions

Meaningful and authentic stakeholder engagement allowed deliberate, iterative, and human-centered co-designing of our implementation-focused initiative. This initiative aims at improving uptake and use of shared access by care partners of older adults, and educational materials are co-designed with the aim of widespread scaling of the initiative. Due to the extensive involvement of our stakeholders, our initiative is well-positioned to reach those aims, and the materials are publicly available to all interested organizations. Following a 12-month demonstration that will include quantitative and qualitative analysis of registration and use of shared access, we will be able to report on the successes and challenges of our initiative.

## Appendix

Multimedia Appendix 1 Correspondence of stakeholder engagements to human-centered design stages.

## Abbreviations

EHR: electronic health record
HCD: human-centered design
PFAC: Patient and Family Advisory Council

## Data Availability

The data sets generated during this study are available from the corresponding author on reasonable request.
