# Supplementary material for: Co-Designing an Initiative to Increase Shared Access to Older Adults’ Patient Portals: Stakeholder Engagement
Source: J Med Internet Res. 2023 Nov 22;25:e46146. doi: 10.2196/46146 (PMC10701652; doi:10.2196/46146)
Supplement: Multimedia Appendix 1 [file jmir_v25i1e46146_app1.docx]

**Multimedia Appendix 1.** Correspondence of stakeholder engagements to human-centered design stages.

**Table S1. Frequency and types of stakeholder engagements corresponding to human-centered design stages**

| **Stakeholder group & number of engagements* with each group** | **Stages of the human-centered design process** | | | | |
| --- | --- | --- | --- | --- | --- |
|  | **Define**  *Figuring out the problem* | **Empathize**  *Understanding people* | **Ideate**  *Generating ideas together* | **Prototype**  *Creating and experimenting* | 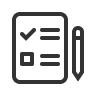  **Test**  *Refining the product* |
| Patients and care partners | 4 | | 1 | | *in the field*  *since July 2022* |
| Clinicians and clinics staff | - | 18 | | 19 |  |
| Medical informatics teams | 4 | | - | - |  |
| Marketing and communication staff | - | - | 2 | 3 |  |
| Clinic leaders and administrators | 5 | - | 2 | 12 |  |
| Thought leaders and funders | 3 | - | 1 | 1 |  |

* in each engagement participated 4-15 people

Our **Advisory Panel members**, alphabetized (membership varied throughout 2020-2022)

| **Rita Choula**, M.A., American Association of Retired Persons | **Cristin Napier**, B.A., Epic Systems Corporation |
| --- | --- |
| **Marie Cleary-Fishman**, M.S., M.B.A., Health Research & Educational Trust | **Jessica Oveys**, B.A., B.B.A., Cerner Corporation |
| **David Dorr**, M.D., M.S., Oregon Health & Science University | **Leslie Pelton**, M.P.A., B.A., Institute for Healthcare Improvement |
| **Margo Edmunds**, Ph.D., AcademyHealth | **Terri Postma**, M.D., Performance-Based Payment Policy Group, Centers for Medicare and Medicaid Services |
| **Elizabeth Palena Hall**, M.I.S., M.B.A., R.N., Office of the National Coordinator for Health Information Technology, U.S. Department of Health and Human Services | **Malek Sayegh**, M.S., Epic Systems Corporation |
| **Kathleen Kelly**, M.P.A., Family Caregiver Alliance | **Jeffery Smith**, M.P.P., American Medical Informatics Association |
| **Hannah Luetke-Stahlman**, M.P.A., Cerner Corporation | **Scott Weinberg**, M.P.A., American Medical Informatics Association and later Health Informatics and Interoperability Group, Office of Burden Reduction and Health Informatics, Centers for Medicare and Medicaid Services |
| **Lana Moriarty**, M.P.H., Office of Policy at Office of the National Coordinator for Health Information Technology |  |
